# Changes in environmental tobacco smoke (ETS) exposure over a 20-year period: cross-sectional and longitudinal analyses

**DOI:** 10.1111/j.1360-0443.2008.02473.x

**Published:** 2009-03

**Authors:** Barbara J Jefferis, Andrew G Thomson, Lucy T Lennon, Colin Feyerabend, Mira Doig, Laura McMeekin, S Goya Wannamethee, Derek G Cook, Peter H Whincup

**Affiliations:** 1 British Regional Heart Study, UCL Department of Primary Care and Population Sciences, Royal Free and University College Medical SchoolLondon, UK,; 2 ABS Laboratories, Wardalls GroveLondon, UK; 3 Division of Community Health Sciences, St George's, University of LondonCranmer Terrace, London, UK

**Keywords:** Cohort study, cotinine, environmental tobacco smoke, tobacco, trend

## Abstract

**Aims:**

To examine long-term changes in environmental tobacco smoke (ETS) exposure in British men between 1978 and 2000, using serum cotinine.

**Design:**

Prospective cohort: British Regional Heart Study.

**Setting:**

General practices in 24 towns in England, Wales and Scotland.

**Participants:**

Non-smoking men: 2125 studied at baseline [questionnaire (Q1): 1978–80, aged 40–59 years], 3046 studied 20 years later (Q20: 1998–2000, aged 60–79 years) and 1208 studied at both times. Non-smokers were men reporting no current smoking with cotinine < 15 ng/ml at Q1 and/or Q20.

**Measurements:**

Serum cotinine to assess ETS exposure.

**Findings:**

In cross-sectional analysis, geometric mean cotinine level declined from 1.36 ng/ml [95% confidence interval (CI): 1.31, 1.42] at Q1 to 0.19 ng/ml (95% CI: 0.18, 0.19) at Q20. The prevalence of cotinine levels ≤ 0.7 ng/ml [associated with low coronary heart disease (CHD) risk] rose from 27.1% at Q1 to 83.3% at Q20. Manual social class and northern region of residence were associated with higher mean cotinine levels both at Q1 and Q20; older age was associated with lower cotinine level at Q20 only. Among 1208 persistent non-smokers, cotinine fell by 1.47 ng/ml (95% CI: 1.37, 1.57), 86% decline. Absolute falls in cotinine were greater in manual occupational groups, in the Midlands and Scotland compared to southern England, although percentage decline was very similar across groups.

**Conclusions:**

A marked decline in ETS exposure occurred in Britain between 1978 and 2000, which is likely to have reduced ETS-related disease risks appreciably before the introduction of legislation banning smoking in public places.

## INTRODUCTION

Environmental tobacco smoke (ETS) exposure is an important public health issue. Numerous epidemiological studies have reported that ETS exposure is associated with elevated risks of lung cancer [1] and cardiovascular diseases in adults [2]–[4] and respiratory problems in children [5]. ETS exposure comprises a mixture of sidestream smoke from burning cigarettes and mainstream smoke exhaled by active smokers. Sidestream smoke may be disproportionately more harmful than mainstream smoke [6]. ETS exposure in non-smoking populations is best quantified by measuring cotinine (a stable metabolite of nicotine measured in serum or saliva, which has a half-life of 16–20 hours, and therefore quantifies recent tobacco exposure [6]).

Between 1978 and 2000 marked changes in factors affecting ETS exposure occurred in the United Kingdom. The prevalence of active smoking in adults fell from 40% to 27% and, among smokers, cigarette consumption declined from 114 to 97 cigarettes/week [7]. Restrictions on smoking both in public places and in work-places increased; by 1997 82% adults reported work-place smoking restrictions [8].

However, information on changes in ETS exposure in the United Kingdom between 1978 and 2000 is very scarce. Evidence is limited to the Health Survey for England (HSE), which reported a small rise in cotinine levels of 0.04 ng/ml (11% increase) in adult non-smokers between 1994 and 1996 [9]. In adolescents a decline in cotinine levels of 0.44 ng/ml (46% fall) was reported between 1988 and 1998 in 11–15-year-olds [10].

We have therefore examined serum cotinine levels and their determinants among non-smoking British men studied in two cross-sectional surveys carried out within a cohort study in 1978–80 and 1998–2000, and the patterns of change in cotinine in a subset of men studied on both occasions 20 years apart. We also examined the proportion of non-smoking men at both time-points who had a cotinine level of ≤ 0.7 ng/ml, a level associated with low coronary heart disease (CHD) risk in our earlier study [4]. In addition, we have examined the influence of socio-demographic and household factors on levels and changes in cotinine levels over this extended period.

## METHODS

The British Regional Heart Study (BRHS) is a prospective study of 7735 men (78% response rate) aged 40–59 years registered with one representative general practice in each of 24 British towns [11]. At entry to the study in 1978–80 [questionnaire 1 (Q1)], nurses administered a health and life-style questionnaire and took blood samples. Men were asked in detail about current and previous smoking history (cigarettes, pipes and cigars), occupation and medication use. Blood samples were taken for all men; in 4735 men in 18 study towns reference samples were stored (−20°C) for subsequent analyses including cotinine. In 1998–2000 (Q20) 4252 of 5699 surviving men (77% response rate), now aged 60–79 years, attended for a further assessment, including a questionnaire survey and the collection of blood samples [11]; cotinine assays were completed in 3900 subjects from all 24 towns. Cotinine measurements at both time-points were available for 2347 men. Baseline serum samples (1978–80) were assayed for cotinine in 2000–01 and follow-up samples (1998–2000) in 2007–08 at the same laboratory (ABS Laboratories Ltd, London, UK). The same method was used in non-smokers on both occasions: a liquid chromatography tandem mass spectrometry (LC MS/MS) assay with a lower limit of detection of 0.02 ng/ml with a limit of quantification of 0.1 ng/ml [12]. No men in the sample reported taking nicotine replacement therapy at Q20 [British National Formulary (BNF) code 4.10][13]. Ethical approval was provided by all relevant local research ethics committees.

Non-smokers were men who reported no current smoking (cigarette, cigar or pipe smoking) and had serum cotinine ≤ 15 ng/ml, as in other literature [9]. Men who reported being non-smokers but with cotinine > 15 ng/m were recoded as smokers (*n* = 58 of 2183 at Q1 and *n* = 58 of 3104 at Q20). Social class was based on longest-held occupation of each man at Q1, classified according to the Registrar General scales: I, II, III non-manual, III manual, IV and V. Men in the armed forces (*n* = 231) were treated as a separate group and were excluded from analyses of trends by social class. Employment status at Q1 and Q20 was coded as employed or not (unemployed plus retired). Region of residence at study entry was defined as Scotland, North, Midlands and South, as in previous studies [14]. At Q20 men recorded their spouse's smoking habits as current, ex-smokers or non-smokers and the number of hours per day that they were exposed to cigarette smoke (i) at home, grouped as none or rarely, 1–5, 6–24 hours; and (ii) elsewhere, grouped as none or rarely, 1–3, 4–24 hours.

### Statistical methods

The distributions of cotinine values (although not changes in cotinine values) were positively skewed, therefore geometric means and 95% confidence intervals (CI) are reported. Cotinine values below the limit of quantification were assigned a value of 0.05 ng/ml, the mid-point between the limit and zero exposure, as in other studies [10].

Geometric mean (95% CI) cotinine levels at Q1 and at Q20 were calculated for each age-group, occupational group and region of residence at study entry, and (at Q20 only) partner's smoking group. Mutually adjusted geometric mean cotinines were then calculated from linear regression models which regressed log cotinine on explanatory variables. Additionally, geometric mean cotinine at Q20 was calculated according to number of hours exposed to cigarette smoke at home, among the men living with a smoker at Q20. To compare the cotinine distribution in non-smokers at Q1 with Q20, natural log cotinine values were plotted against age for both surveys.

The intra-individual absolute change in cotinine level between the two surveys was calculated as (cotinine Q20–cotinine Q1) in the 1208 non-smokers with cotinine data at both surveys. It was approximately normally distributed and was modelled using linear regression in relation to covariates including age at study entry, occupational position, region of residence and partner's smoking habit. Additionally, the percentage change between adjusted geometric means, calculated as 100 × [(Q20 − Q1)/Q1], was reported for each level of the covariates.

## RESULTS

Baseline (Q1) analyses are based on 4636 men with both questionnaire smoking history and cotinine levels in 1978–80, of whom 2125 (46%) were non-smokers. Follow-up (Q20) analyses are based on 3784 men with both questionnaire smoking history and cotinine levels in 1998–2000, of whom 3046 (81%) were non-smokers. Questionnaire smoking history and cotinine data were available at both time-points on 2272 men; of these, 1208 (53%) were non-smokers on both occasions.

### Cross sectional analysis

**Figure 1:**
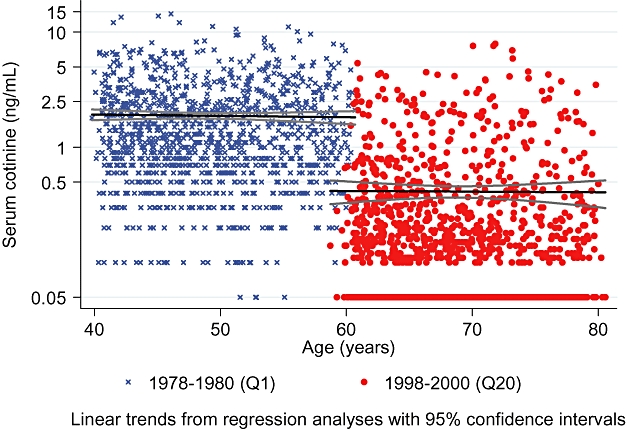
Association between serum cotinine and age in non-smokers at questionnaire 1 (Q1) (1978–80) and Q20 (1998–2000)

In cross-sectional analyses at the two time-points, geometric mean cotinine was 1.36 ng/ml (95% CI: 1.31, 1.42) in 1978–80 and 0.19 ng/ml (95% CI: 0.18, 0.19) in 1998–2000, a decline of 1.17 ng/ml (86%). Figure 1 illustrates the cotinine levels in non-smokers in relation to age at Q1 and Q20. There was a marked decline in cotinine levels between the two surveys; at Q1 cotinine did not vary with age, but at Q20 there was a weak inverse association between cotinine and age. The distribution of exceptionally low cotinine values also increased markedly between the two time-points. At Q1 only 0.5% (95% CI: 0.3, 0.9) (*n* = 11/2125) of men had cotinine levels less than or equal to the level of detection, compared to 35.1% (95% CI: 33.4, 36.8) (1069/3046) at Q20. The proportion of men with a cotinine level of ≤0.7 ng/ml increased from 27.1% (95% CI: 25.2, 29.0) (575/2125) at Q1 to 83.3% (95% CI: 81.9, 84.6) (2536/3046) at Q20.

The socio-demographic correlates of cotinine in non-smokers were examined at both time-points (Table 1). Age at Q1 (40–59 years) was not associated with cotinine at Q1, but at Q20 (60–79 years) younger men had higher mean cotinine levels. Men in manual occupational groups, in employment or living in northern England had higher mean cotinine levels, both at Q1 and at Q20. Mutual adjustment for age, class, employment status and region did not change materially the group means or linear trends. At Q20, additional information was available about spouse smoking habits; men who lived with current smokers had almost eight times higher adjusted geometric mean cotinine values than men who lived with non-smokers [1.19 ng/ml (95% CI: 1.04, 1.34) versus 0.15 ng/ml (95% CI: 0.14, 0.15), respectively]. Men also reported the number of hours per day that they were exposed to tobacco smoke in the home. Among non-smokers who lived with a smoker, exposure to smoke for 1–5 hours per day was associated with cotinine levels double that of with men reporting little or no exposure and nearly 10 times that of men who did not live with a smoker [1.39 ng/ml (95% CI: 1.15, 1.69) versus 0.67 ng/ml (95% CI: 0.53, 0.86) versus 0.15 ng/ml (95% CI: 0.15, 0.16), respectively]. There was evidence for dose-dependent associations of increasing cotinine with increasing ETS exposure both at home and elsewhere. Reporting more than 1 hour of exposure to smoke elsewhere was associated with more than double the cotinine levels in men with none/rare exposure. The adjustment for age, social class, employment and region attenuated the mean values slightly. Among non-smokers with cotinine > 0.7 ng/ml and data on self-reported exposure at Q20, 50% lived with a partner who smoked; of these 80% reported ≥ 1 hour/day exposure to ETS at home and 40% reported ≥ 1 hour/day exposure ETS elsewhere than at home (data not presented).

**Table 1:** Adjusted geometric mean [95% confidence interval (CI)] cotinine level in non smokers at (i) questionnaire 1 (Q1) and (ii) Q20 by demographic factors.

	*n (%)*	*Cotinine at Q1 (age 38–61 years)^a^*	*n (%)*	*Cotinine at Q20 (age 58–81 years)*^a^
Grand mean	2125 (100)	1.36 (1.31, 1.42)	3046 (100)	0.19 (0.18, 0.19)
Age (years)				
Q1: 38–44, Q20: 58–64	516 (24.3)	1.40 (1.29, 1.52)	930 (30.5)	0.20 (0.18, 0.21)
Q1: 45–49, Q20: 65–69	519 (24.4)	1.27 (1.17, 1.38)	869 (28.5)	0.19 (0.18, 0.20)
Q1: 50–54, Q20: 70–74	524 (24.7)	1.39 (1.29, 1.51)	718 (23.6)	0.19 (0.17, 0.20)
Q1: 55–61, Q20: 75–81	566 (26.6)	1.39 (1.29, 1.51)	529 (17.4)	0.17 (0.15, 0.18)
*P*(trend)^b^		0.665		< 0.001
Class at Q1				
I	244 (11.5)	1.03 (0.92, 1.16)	313 (10.3)	0.15 (0.14, 0.17)
II	536 (25.2)	1.22 (1.13, 1.32)	849 (27.9)	0.16 (0.15, 0.18)
III NM	197 (9.3)	1.20 (1.05, 1.37)	313 (10.3)	0.17 (0.15, 0.19)
III M	79 (3.7)	1.80 (1.46, 2.23)	89 (2.9)	0.25 (0.20, 0.31)
IV M	850 (40.0)	1.49 (1.39, 1.59)	1169 (38.4)	0.21 (0.20, 0.22)
V M	179 (1.9)	1.76 (1.53, 2.02)	242 (7.9)	0.21 (0.18, 0.24)
Armed forces	40 (1.9)	1.77 (1.32, 2.37)	71 (2.3)	0.21 (0.17, 0.27)
*P*(trend)^b^		< 0.001		< 0.001
Employment (at Q1, at Q20)				
Employed	2033 (95.7)	1.38 (1.32, 1.44)	226 (18.7)	0.20 (0.18, 0.22)
Not employed	92 (4.3)	1.07 (0.88, 1.30)	939 (77.8)	0.18 (0.18, 0.19)
Missing	0 (0)		99 (2.3)	0.16 (0.12, 0.21)
*P*(trend)^b^		0.027		0.006
Region at Q1				
South	780 (36.7)	1.04 (0.97, 1.11)	1076 (35.5)	0.15 (0.15, 0.17)
Midlands	104 (4.9)	1.69 (1.41, 2.03)	479 (15.7)	0.19 (0.18, 0.21)
North	877 (41.3)	1.49 (1.40, 1.58)	1171 (38.4)	0.20 (0.19, 0.21)
Scotland	364 (17.1)	1.88 (1.70, 2.07)	320 (10.5)	0.24 (0.21, 0.26)
*P*(trend)^b^		< 0.001		< 0.001
Spouse smoking at Q20				
Non-smoker			2262 (74.3)	0.15 (0.14, 0.15)
Ex-smoker			226 (7.4)	0.19 (0.17, 0.22)
Smoker			275 (9.0)	1.19 (1.04, 1.34)
NA (no spouse)			168 (5.5)	0.19 (0.17, 0.23)
Missing			115 (3.8)	0.18 (0.14, 0.22)
*P*(trend)^b^				< 0.001
Hours/day exposed to ETS at home at Q20				
None/rare			2764 (90.9)	0.15 (0.15, 0.16)^c^
None/rare and partner smokes			73 (2.4)	0.67 (0.53, 0.86)^c^
1–5			118 (3.9)	1.39 (1.15, 1.69)^c^
6–24			64 (2.1)	1.83 (1.40, 2.38)^c^
Missing			21 (0.7)	0.95 (0.60, 1.50)^c^
*P*(trend)^b^				< 0.001
Hours/day exposed to ETS elsewhere at Q20				
None/rare			2335 (76.7)	0.16 (0.15, 0.17)
1–3			223 (7.3)	0.38 (0.33, 0.43)
4–24			80 (2.7)	0.47 (0.37, 0.59)
Missing			408 (13.4)	0.25 (0.22, 0.28)
*P*(trend)^b^				< 0.001

a Cotinine at Q1 adjusted for age, class, employment status and region; cotinine at Q20 adjusted for age, class, employment status, region, spouse smoking and hours/day exposed to environmental tobacco smoke (ETS) outside home;

b trend tests from separate linear regression models. Missing group/armed forces excluded from test;

c adjusted model includes age, class, employment status, region, hours/day exposed to ETS at home and elsewhere. NA: not applicable.

### Longitudinal analysis

Cotinine levels were investigated in 1208 persistent non-smokers with cotinine < 15 ng/ml at both Q1 and Q20. The geometric mean cotinine levels of these men were were 1.23 ng/ml (95% CI: 1.17, 1.30) at Q1 and 0.16 ng/ml (0.15, 0.17) at Q20. These values were slightly lower than those in the two cross-sectional surveys as a whole (1.36 ng/ml at Q1, 0.19 ng/ml at Q20), although the percentage decline in the longitudinal population (87%) was nearly identical to that in the two cross-sectional surveys (86%). The longitudinal sample contained a higher proportion of professional/managerial workers (40.3%) than did the longitudinal sample of non-smokers (36.7%). In the 1208 non-smoking men studied both at Q1 and Q20, baseline and follow-up cotinine values were correlated moderately (*r* = 0.35). The absolute change in cotinine level and its determinants are presented in Table 2. The size of decline was unrelated to age, but was greater among men in manual compared to non-manual occupational groups and in men in the Midlands and Scotland compared to southern England; these falls were little affected by mutual adjustment. The overall proportional fall in cotinine (87%) was, however, very similar in all age-groups, social classes and regions (Table 2). Thus, although absolute falls in cotinine level were greater in men from social class V (manual) than social class I (professional) (2.10 ng/ml compared with 1.13 ng/ml, respectively), proportional falls were very similar (89% and 87%, respectively).

**Table 2:** Changes in cotinine level (expressed as mean and proportion) between 1978–80 and 1998–2000 among non-smokers: influence of age, social class and region (*n* = 1208).

	*Non smokers Q1–Q20*		
	*Unadjusted cotinine change Q1–Q20*	*Mutually adjusted cotinine change Q1–Q20*^b^	*% change Q1–Q20*^c^
Grand mean	−1.47 (−1.57, −1.37)	−1.47 (−1.56, −1.37)	−87
Age (years) at Q1			
38–44	−1.52 (−1.71, −1.34)	−1.50 (−1.68, −1.31)	−86
45–49	−1.46 (−1.65, −1.28)	−1.49 (−1.67, −1.31)	−88
50–54	−1.35 (−1.56, −1.15)	−1.37 (−1.57, −1.17)	−85
55–61	−1.53 (−1.76, −1.30)	−1.52 (−1.74, −1.30)	−89
*P*(trend)^a^	0.735	0.740	
Class at Q1			
I and II	−1.02 (−1.29, −0.76)	−1.13 (−1.39, −0.86)	−87
II	−1.36 (−1.55, −1.17)	−1.37 (−1.55, −1.18)	−88
III NM	−1.34 (−1.65, −1.04)	−1.36 (−1.66, −1.05)	−89
III M	−1.93 (−2.51, −1.35)	−1.83 (−2.41, −1.25)	−88
IV M	−1.58 (−1.74, −1.42)	−1.54 (−1.70, −1.38)	−85
V M	−2.13 (−2.49, −1.78)	−2.10 (−2.46, −1.75)	−89
Armed forces	−1.22 (−1.95, −0.50)	−1.32 (−2.05, −0.61)	−87
*P*(trend)^a^	<0.001	<0.001	
Region at Q1			
South	−1.17 (−1.32, −1.01)	−1.22 (−1.37, −1.06)	−87
Midlands	−1.87 (−2.13, −1.61)	−2.10 (−2.60, −1.60)	−93
North	−1.59 (−1.74, −1.43)	−1.55 (−1.70, −1.39)	−86
Scotland	−1.75 (−1.99, −1.50)	−1.73 (−1.97, −1.49)	−87
*P*(trend)^a^	< 0.001	< 0.001	

a Trend tests all from separate linear regression models;

b means adjusted for age, social class and region;

c % change calculated as [adjusted geometric mean questionnaire 1 (Q1)—adjusted geometric mean Q20/adjusted geometric mean Q1] in 1208 men who were non-smokers at Q1 and Q20. Means adjusted for age, region and class only.

All analyses were repeated using a more recently recommended and more conservative cotinine threshold of 9.5 ng/ml [21] to identify non-smokers, and this did not affect the results materially.

## DISCUSSION

In this study of middle-aged and older British men, a marked decline in ETS exposure occurred between 1978 and 2000, with mean cotinine levels falling by 86% from 1.36 ng/ml to 0.19 ng/ml. The prevalence of cotinine levels sufficiently high to cause adverse cardiovascular consequences > 0.7 ng/ml [5] fell from 83% to 27%. Social class and region were related independently to cotinine levels both in 1978 and in 2000. The men with the highest levels of exposure in 1978 (i.e. men in more manual occupations and those living further north in the United Kingdom) experienced the greatest absolute decline in exposure up to 2000, although the percentage changes were similar.

### Comparison of cotinine levels with other data

There are limited data on cotinine levels in British adults in the 1978–2000 period. In the Scottish Heart Health Study (SHHS), median serum cotinine in all male non-smokers aged 40–59 years studied in 1984–86 was 0.68 ng/ml [15], somewhat lower than the levels in the BRHS in 1978–80 (1.36 ng/ml). In the national HSE, geometric mean plasma cotinine in 1994–96 combined was 0.42 ng/ml [9], about twice the level observed in BRHS in 1998–2000 (0.19 ng/ml). The decline seen in BRHS data is consistent with a report based on salivary cotinine measurements (which estimates cotinine levels about 25% higher than those in blood [16]) in the HSE and Scottish Health Survey, in which mean salivary cotinine levels in adults with a non-smoking partner fell by slightly more than a half between 1993 [0.58 ng/ml (95% CI: 0.56–0.61)] and 2003 [0.25 (95%: CI 0.23–0.27)], although declines were not evident in homes where adults smoked [17]. The proportion of adults with cotinine levels below detectable levels has also increased over the study period: in SHHS in 1984–86 more than one-quarter of non-smoking participants had no measurable cotinine level, compared with 0.5% men in BRHS about 6 years earlier. This evidence, although incomplete, suggests that the marked secular decline observed in BRHS has probably taken place gradually during the 1980s and 1990s; this would be consistent with the secular decline observed in salivary cotinine levels in British school children, in whom levels fell gradually between 1988 and 1998, from 0.96 mg/nl to 0.52 (0.43, 0.62) [10]. In the National Health and Nutrition Examination Study (NHANES) study in the United States, broadly similar patterns are seen. Between 1988–91 and 2001–2, cotinine levels in non-smoking adults (aged over 20 years) fell by 70% to a level of 0.035 ng/ml (95% CI: 0.035, 0.060), lower than that seen in BRHS. The prevalence of undetectable cotinine levels rose from 12% in 1988–91 to 67% in 2001–02 [18].

Manual social groups and northern regions had the highest initial levels and greatest absolute decline in cotinine in BRHS. Similar cross-sectional relations between social class, region and cotinine levels have been reported in HSE [9]. Similar differences in patterns of decline between different social class groups were observed in NHANES data in the United States over a shorter time-period (1988–2002) [19]. These patterns and differences almost certainly reflect the social class and regional differences in prevalence of cigarette smoking and subsequent changes in smoking prevalence [8]. The higher cotinine levels of non-smokers living with a smoker in BRHS in 1998–2000 are broadly consistent with 1994–96 HSE data and 1996–2003 HSE/Scottish Health Survey data. In BRHS, cotinine levels were nearly eight times higher among men living with a partner who smoked compared to a non-smoker; 1.19 ng/ml (1.04, 1.34) compared to 0.15 ng/ml (0.14, 0.15). In HSE, non-smoking adults cohabiting with another non-smoker had adjusted mean serum cotinine levels of 0.34 ng/ml; own cotinine level increased with increasing partner's cigarette consumption (up to 3.5-fold for smokers of > 30/day) [9]. In combined HSE/Scottish Health Survey data, salivary cotinine was nearly five times greater in adults in a smoking home compared to adults from a non-smoking home: 1.46 ng/ml compared to 0.31 ng/ml [17].

### Strengths and weaknesses

The present study is unique in quantifying changes in ETS exposure in British men over an extended (20 years) period using an objective biomarker (cotinine) to measure exposure. The use of serum cotinine rather than self-report to quantify ETS exposure is a considerable strength. It is well validated for this purpose [6] and is stable over extended periods, so levels are unlikely to have been affected by long-term storage. Although there is genetic variation in cotinine metabolism [20], this would not be expected to affect the intra-individual changes in cotinine levels. Most other studies have been based on much shorter time-periods. Although there were appreciable losses to follow-up during the study, the assessments of changes in ETS exposure and its determinants are unlikely to have been influenced seriously by selection bias. Bias is unlikely, given that the percentage decline in cotinine in the longitudinal sample was almost the same as the change in the cross-sectional samples and, further, that baseline (Q1) cotinine levels of non-smokers who survived to Q20 were only slightly lower than in non-smokers who did not survive to Q20: 1.51 ng/ml (95% CI: 1.44, 1.59) compared to 1.85 (95% CI: 1.63, 2.09). Moreover, under-representation of men from manual social classes in the longitudinal analyses was slight. However, the inverse association between age and cotinine level in the follow-up study, suggesting that men aged 75–81 years may have systematically lower ETS exposure than younger men, raises the possibility that the overall decline in exposure has been slightly overestimated by including these older men. However, even for the oldest age-group, the proportional decline in cotinine is only slightly higher (89%) than in the youngest age-group (86%). Although we study men, overall patterns are likely to be very similar in women, although ETS exposure is likely to have been slightly lower in women. Other studies reported lower levels of cotinine exposure in non-smoking women than in non-smoking men [9],[18]. The sensitivity analysis demonstrated the robustness of the results using a more conservative cotinine threshold to identify non-smokers [21].

### Implications of the results

The results show clearly that there was a very marked decline in ETS exposures among non-smokers in Britain between 1978 and 2000, well before the legislative bans on smoking in public places implemented in England, Scotland and Wales between 2006 and 2007. This decline was probably gradual and is likely to have had several causes, although their relative importance is difficult to assess. First, the prevalence of active smokers declined during this period, so the men were exposed to smoke from fewer people. By 2000, only 14% of men in the study were cigarette smokers (compared with 54% in 1978–80) and only 13% lived with a partner who currently smoked. Secondly, the number of cigarettes smoked by active smokers has declined. Both trends are expected to affect ETS exposure of non-smoking adults at home and at leisure. Thirdly, the period covered by this study includes increasing restrictions on smoking, particularly in the work-place, with 82% adults reporting work-place smoking restrictions by 1997 [8]. The results do not suggest that ageing or retirement of the cohort underlies the observed decline in cotinine levels. Age was not associated with cotinine at Q1 and only weakly at Q20 (a difference of 0.03 ng/ml across the 23-year age range). At both Q1 and Q20 men in employment had higher cotinine levels than unemployed or retired men. However, the difference in mean cotinine levels by employment status (0.31 ng/ml at Q1 and 0.02 ng/ml at Q20) is much smaller than the difference in grand means (1.17 ng/ml) over the 20-year period, suggesting that ageing and retirement were not major determinants of observed cotinine decline over the study period.

The impact of the decline in ETS exposure on adult health is difficult to quantify. Although measurement of cotinine allows accurate quantification of ETS exposure, data on the relationship of cotinine levels to health outcomes among non-smokers are limited. In a previous study, we showed that in non-smokers cotinine levels above 0.7 ng/ml at Q1 were associated with elevated risks of CHD over a 20-year period [4]. The marked decline in average cotinine levels observed between 1978 and 2000 was accompanied by a very marked decline in the proportion of non-smokers with cotinine levels above 0.7 ng/ml, which declined from 83% in 1978–80 to 27% in 1998–2000. On the basis of our earlier report on the relations of cotinine exposure to CHD risk [4], the decline in the proportion of subjects with a cotinine level of more than 0.7 ng/ml could have accounted for a decline in CHD risk of about 20% over the study period. However, it is possible that the rapid decline in ETS exposure led us to underestimate the risks of CHD associated with ETS exposure, a point we made in our earlier paper [4], in which case the contribution of declining ETS exposure to the documented decline in CHD risk [22] may also have been underestimated. However, studies linking cotinine level with intermediate vascular risk markers have suggested that levels of cotinine as low as 0.2 ng/ml could be associated with increased CHD risk [23]. If this were the case, it would suggest that, although substantial reductions in cotinine-related risk occurred between 1978 and 2000, further reductions in cotinine levels would be necessary to abolish associated health risks completely.

It is likely that over the 20-year period there has been a shift in the balance of ETS exposure sources. At Q1 a substantial proportion of the ETS exposure arose outside the home, in the work-place and other public spaces including pubs and restaurants, although during the 20-year follow-up exposure in the work-place was removed progressively, particularly in this older population who are mainly retired and have left the work-place. The important role of the home as a continuing source of ETS exposure is emphasized by the 1998–2000 BRHS survey, in which half the non-smokers with cotinine > 0.7 ng/ml lived with a partner who smoked. There was also a strong association between hours of domestic exposure and cotinine levels among men living with smokers; average cotinine levels in men living with a smoker were similar to those of all non-smoking men 20 years previously. Although this population, being retired, may overestimate the relevance of domestic exposure for the whole population, home exposure is likely to be the dominant exposure source. This situation is likely to have been reinforced by the subsequent national bans on smoking in public places, which by reducing ETS exposure in public places and work-places [24] emphasizes the relative importance of domestic exposure as a source of residual ETS exposure. Importantly, it is not thought that domestic exposure within smoking households rises in response to public smoking bans [25]. After the smoking ban in Scotland, cotinine levels remained stable among those living in households with smokers, while levels declined among non-smoking adults living in non-smoking households [24].

Our present and previous studies suggest that there is potential for further health gain from additional reductions in ETS exposure, aiming particularly to reduce further the prevalence of non-smoking subjects with a cotinine level of > 0.7 ng/ml. Such reductions are likely to depend upon further reducing domestic ETS exposure.

## CONCLUSIONS

Substantial declines in ETS exposure were observed between 1978 and 2000, which probably reflect a steady decline over the period and are likely to have had appreciable effects on CHD risk. The decline in exposure probably reflects decreased smoking prevalence and amounts smoked, and restrictions on smoking in work-places. Further reductions in ETS exposure are likely to require greater efforts to limit exposure in the home.

## References

[1] Taylor R, Najafi F, Dobson A (2007). Meta-analysis of studies of passive smoking and lung cancer: effects of study type and continent. Int J Epidemiol.

[2] He J, Vupputuri S, Allen K, Prerost MR, Hughes J, Whelton PK (1999). Passive smoking and the risk of coronary heart disease—a meta-analysis of epidemiologic studies. N Engl J Med.

[3] Law MR, Morris JK, Wald NJ (1997). Environmental tobacco smoke exposure and ischaemic heart disease: an evaluation of the evidence. BMJ.

[4] Whincup PH, Gilg JA, Emberson JR, Jarvis MJ, Feyerabend C, Bryant A (2004). Passive smoking and risk of coronary heart disease and stroke: prospective study with cotinine measurement. BMJ.

[5] Cook DG, Strachan DP (1999). Health effects of passive smoking-10: summary of effects of parental smoking on the respiratory health of children and implications for research. Thorax.

[6] Benowitz NL (1996). Cotinine as a biomarker of environmental tobacco smoke exposure. Epidemiol Rev.

[7] Walker A, Maher J, Coulthard M, Goddard E, Thomas M (2001). Smoking. Living in Britain. Results from the 2000/2001 General Household Survey.

[8] Department of Health (2000). Statistics on Smoking: England, 1978 Onwards.

[9] Jarvis MJ, Feyerabend C, Bryant A, Hedges B, Primatesta P (2001). Passive smoking in the home: plasma cotinine concentrations in non-smokers with smoking partners. Tob Control.

[10] Jarvis MJ, Goddard E, Higgins V, Feyerabend C, Bryant A, Cook DG (2000). Children's exposure to passive smoking in England since the 1980s: cotinine evidence from population surveys. BMJ.

[11] Walker M, Whincup PH, Shaper AG (2004). The British Regional Heart Study 1975–2004. Int J Epidemiol.

[12] Feyerabend C, Russell MA (1990). A rapid gas–liquid chromatographic method for the determination of cotinine and nicotine in biological fluids. J Pharm Pharmacol.

[13] Joint Formulary Committee (1999). British National Formulary.

[14] Elford J, Phillips A, Thomson AG, Shaper AG (1990). Migration and geographic variations in blood pressure in Britain. BMJ.

[15] Tunstall-Pedoe H, Woodward M, Brown CA (1991). Tea drinking, passive smoking, smoking deception and serum cotinine in the Scottish Heart Health Study. J Clin Epidemiol.

[16] Jarvis MJ, Primatesta P, Erens B, Feyerabend C, Bryant A (2003). Measuring nicotine intake in population surveys: comparability of saliva cotinine and plasma cotinine estimates. Nicotine Tob Res.

[17] Tobacco Advisory Group of the Royal College of Physicians (2005). Going Smoke-Free.

[18] Pirkle JL, Bernert JT, Caudill SP, Sosnoff CS, Pechacek TF (2006). Trends in the exposure of nonsmokers in the U.S. population to secondhand smoke: 1988–2002. Environ Health Perspect.

[19] Arheart KL, Lee DJ, Dietz NA, Wilkinson JD, Clark JD, LeBlanc WG (2008). Declining trends in serum cotinine levels in US worker groups: the power of policy. J Occup Environ Med.

[20] Benowitz NL (2008). Clinical pharmacology of nicotine: implications for understanding, preventing, and treating tobacco addiction. Clin Pharmacol Ther.

[21] Jarvis MJ, Fidler J, Mindell J, Feyerabend C, West R (2008). Assessing smoking status in children, adolescents and adults: cotinine cut-points revisited. Addiction.

[22] Lampe FC, Morris RW, Walker M, Shaper AG, Whincup PH (2005). Trends in rates of different forms of diagnosed coronary heart disease, 1978 to 2000: prospective, population based study of British men. BMJ.

[23] Venn A, Britton J (2007). Exposure to secondhand smoke and biomarkers of cardiovascular disease risk in never-smoking adults. Circulation.

[24] Haw SJ, Gruer L (2007). Changes in exposure of adult non-smokers to secondhand smoke after implementation of smoke-free legislation in Scotland: national cross sectional survey. BMJ.

[25] Pierce JP, Leon ME (2008). Effectiveness of smoke-free policies. Lancet Oncol.

